# *In situ* RT-PCR Optimized for Electron Microscopy Allows Description of Subcellular Morphology of Target mRNA-Expressing Cells in the Brain

**DOI:** 10.3389/fncel.2017.00141

**Published:** 2017-05-16

**Authors:** Laura Cubas-Nuñez, María Duran-Moreno, Jessica Castillo-Villalba, Jorge Fuentes-Maestre, Bonaventura Casanova, José M. García-Verdugo, Sara Gil-Perotín

**Affiliations:** ^1^Multiple Sclerosis and Neural Regeneration Research Group, Fundación para la Investigación La Fe, Hospital Universitario y Politécnico La FeValencia, Spain; ^2^Comparative Neurobiology Department, Instituto Cavanilles de Biodiversidad y Biología EvolutivaValencia, Spain; ^3^Neurology Department, Hospital Universitario y Politécnico La FeValencia, Spain

**Keywords:** gene expression, brain, electron microscopy, immunogold, epoxy resin, pre-embedding, biotin-dUTP, TEM

## Abstract

*In situ* RT-PCR detects and amplifies mRNA (cDNA) while obtaining spatial information of gene expression. When the intended use is an ultrastructural analysis of morphology, the procedure may be technically challenging and quality of tissue dramatically altered by proteolytic digestion and extreme astringency and temperature conditions. We describe a low-damaging protocol of *in situ* RT-PCR combined to conventional electron microscopy that preserves fine morphology, increases sensitivity, and decreases costs and complexity associated to RNA probes.

## Introduction

*In situ* hybridization (ISH) specifically detects mRNA targets and obtains temporal and spatial information of gene expression by means of hybridization with a RNA or, alternatively a DNA probe (Uehara et al., 1993; Nuovo, 2010). The lack of sensitivity to detect low expression of mRNA, the complexity and high expenses of probe design, and the need to set up protocols individually for each probe makes ISH a long, not easily reproducible, discouraging method. In contrast, *in situ* RT-PCR is performed directly on the tissue and comprises a combination of reverse transcription of mRNA into cDNA, polymerase chain reaction (PCR) of cDNA templates with specific primers and labeled dNTPs, and, finally, immunodetection of the PCR product. It has the added advantage of providing higher sensitivities than ISH, secondary to amplification. Both ISH and RT-PCR have applications to electron microscopy, but combination of mRNA detection with fine morphological analyses is a difficult task (Le Guellec and Frappart, 1993; Le Guellec, 1998; Morel et al., 1998; Cmarko et al., 2014). Pre-embedding methods achieve high signal-to-noise ratios but require longer proteolytic digestion times (tissue-damaging), and result in increased mispriming rates that are detected as an unspecific signal in the nucleus (Cmarko et al., 2014). Post-embedding detection is preferred and often performed on samples embedded in acrylic resins (Lowycril K4M, LR-White) that polymerize by UV light or low temperature, so nucleic acids are preserved. The main drawbacks of acrylic resins are the need for specific equipment and trained personal for the embedding process, with low image quality results and structurally poorly defined cellular structures. Acrylic resins also exhibit less stability under the electron beam compared to conventional epoxy resins (Le Guellec, 1998).

**Figure 1:**
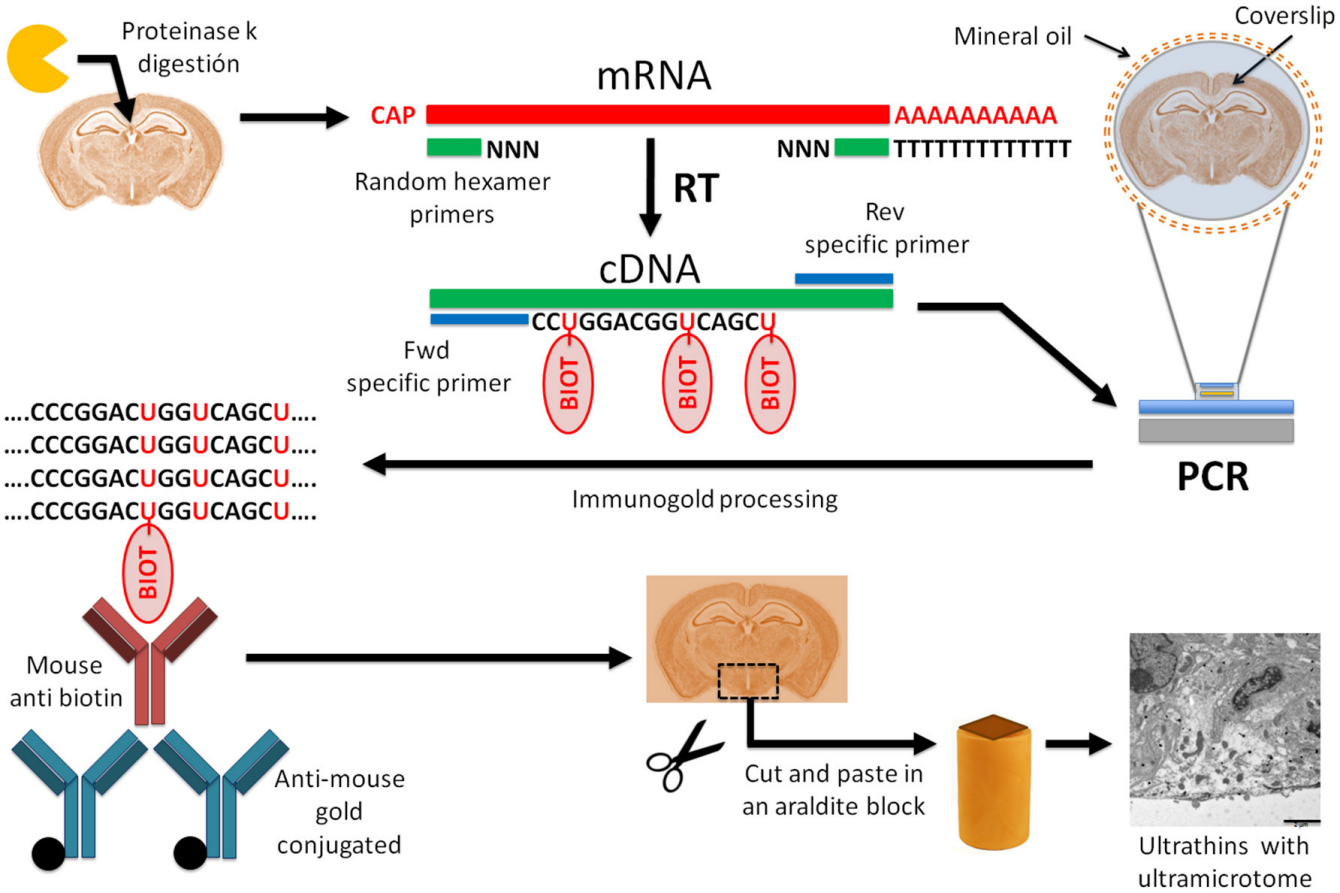
**Diagram of the protocol of ***in situ*** RT-PCR combined with immunogold labeling for electron microscopy**. Schematic diagram of *in situ* RT-PCR immunogold protocol. Tissue digestion with proteinase K increases the nucleic acid accessibility. After proteinase K treatment, we performed reverse transcription of mRNA to cDNA followed by a polymerase chain reaction with specific primers to amplify the gene of interest. In this step, biotin-labeled nucleotides are added to the PCR mix and incorporated into the reaction product. For this step we used a thermocycler adapted to glass slides. Then, PCR product was fixed with 4% PFA/0.5%GA, followed by immunogold labeling against biotin with gold-conjugated antibodies. After embedding the tissue in epoxy resin with conventional protocols, we obtained ultrathin sections with an ultramicrotome and detected gold particles with electron microscopy.

Here, we report an easy, reproducible, and low-damaging *in situ* RT-PCR immunogold staining protocol for ultrastructural mRNA expression studies that overcomes the lack of specificity and sensitivity of protein and mRNA detection, respectively. We used it to detect vimentin mRNA expression in ependymal cells of the third ventricle (3V) in the adult mouse brain but it is a suitable method for any mRNA and any brain region. Our procedure could be splitted into several stages (diagram in Figure 1): proteinase K digestion, mRNA reverse transcription into cDNA, mRNA-specific PCR with labeled nucleotides, immunogold labeling, and tissue embedding in araldite to, finally obtain ultrathin sections for observation under TEM.

## Materials and methods

### Sample preparation

Adult mice, *N* = 6, C57BL (P60) were perfused with a 4% paraformaldehyde (PFA)-0.5% glutaraldehyde (GA) solution and preserved in 0.1 M PB with 0.05% sodium azide. Adult mouse brains were post-fixed with 4% paraformaldehyde (PFA)-0.5% GA and 200 μm coronal sections were obtained with a vibratome (Vibratome VT1000S; Leica Microsystems, Wetzlar, Germany). The optimal thickness was set to 200 μm in order to preserve morphology upon proteolysis and exposure to high temperatures. We opted for 4% PFA with low GA concentration (0.5%) because protein cross-linking was weaker than with 2.5% GA in terms of preventing penetration of primers or probes. All the procedures had been previously evaluated and approved by the Ethical committee of the University of Valencia and the government of Valencia (#A1365526174622) following the current legislation of the European Commission (Directive 2010/63/EU).

### Tissue permeabilization

Proteinase K (20 mg/ml Ambion-Life Technologies, Carlsbad, USA) was used to permeabilize cell membranes. Proteinase K has a 100% activity at 37°C and 80% at room temperature. We tested different concentrations of the enzyme and different times of exposition on mouse vibratome sections of diverse thickness. We determined that 200 μm sections preserved their ultrastructural morphology upon 10 min of proteinase K (5 μg/ml) digestion at room temperature. To prepare the solution of the enzyme, the stock was diluted in DEPC-water (#46-2224, Invitrogen-Thermo Fisher, Waltham, MA). Sections were mounted on slides during this step and washed with DEPC-PB and DEPC-water.

### Reverse transcription and polymerase chain reaction

#### Reverse transcription

Reverse transcription to convert mRNA into cDNA was performed using SuperScriptTM II RT. We placed tissue slices on a glass slide and added reagents on top of the tissue. First, tissue was covered with a drop containing random primers (#N8080127, Invitrogen) and dNTP mix (10 mM, #Y02256, Invitrogen) and completely covered with a 1 cm^2^ piece of Parafilm® (Sigma-Aldrich, St. Louis, MO, USA). Mixture was heated at 65°C for 5 min, and then quickly chilled on ice. Afterwards, we added 5X first strand buffer (#Y02321, Invitrogen), 0.1 M DTT (#Y00122, Invitrogen) and RNase OUT, a protease inhibitor (40 U/μL) (#10777-019, Invitrogen), and reaction temperature was set at 25°C for 2 min. Reverse transcriptase (#18064-022, Invitrogen) was added to the reaction mixture (Table 1) on the surface of the tissue and incubated sequentially at 25°C for 10 min, 42°C for 50 min (to activate the reaction), and 70°C for 15 min (to inactivate the enzyme). The reaction product was tempered at 20°C for 20 min. This step concluded with several washes of 0.1 M PB.

**Table 1:** **Reagents used for RT-PCR in a single section**.

**Reagents for reverse transcription**	**Volume (μL)**
Random primers	3
dNTP mix 10 mM	3
ddH_2_O	36
5X First strand buffer	12
DTT 0.1 M	6
RNase OUT 40 U/μL	3
RT enzyme	1
Total volume	64
**Reagents for PCR**	**Volume (μL)**
10X PCR buffer with MgCl2	2.5
dNTP mix 10 mM	0.5
Fwd primer 10 μM	2
Rev primer 10 μM	2
Taq DNA polimerase (5U/μL)	0.5
Biotinylated dUTP	0.6
Autoclaved ddH_2_O	16.9
Total volume	25

#### Primer design

mRNA sequence of interest (GenBank: BC089335.1) was found in the NCBI database (http://www.ncbi.nlm.nih.gov) and boundaries between exons and introns were determined comparing mRNA sequence with genomic mouse DNA using Splign (http://www.ncbi.nlm.nih.gov/sutils/splign/splign.cgi) and UCSC Browse (www.genome.ucsc.edu). A pair of primers was obtained with Primer3 (http://www.primer3.ut.ee), introducing cDNA sequence, and conditioning the search to primers that overlapped between exons, and with a minimal length of 450 pair of bases (pb) (fwd: GCGAGAGAAATTGCAGGAGG; Tm = 60 and rev: ACTCGTTAGTGCCTTTAAGGG; Tm = 56). The absence of secondary structures at working temperature was tested with specific software (GeneRunner, Hastings Software Inc., Hastings, NY, USA; http://www.generunner.net). To rule out that primers did not amplify gDNA, an online tool, was used, UCSC *In-silico* PCR (http://www.genome.ucsc.edu/cgi-bin/hgPcr) and then, a Blast search was performed (http://www.blast.ncbi.nlm.nih.gov/Blast.cgi) to look for homologous sequences and to verify that selected primers amplified vimentin sequence.

#### Polymerase chain reaction

A gradient PCR was performed using the primers and mouse striatum cDNA to test distinct melting temperatures (Tm = 56, 58, and 60°C) in a conventional thermocycler (Mastercycler, Eppendorf, Hamburg, Germany). To perform the *in situ* PCR, we used a thermal cycler adapted for glass slides, AmpliSpeed® (Beckman Coulter, Brea, CA, USA), that consists of a rectangular surface 75 × 26 mm made of conductive material that is homogeneously heated. This surface is embedded in a metal support and has a propylene cover that lets the vapor exit. The slide was set on the thermocycler surface with the tissue section above and a sealing system to avoid the evaporation of the reagents added over the tissue that consisted of a circular glass coverslip and mineral oil (#M5904, Sigma). PCR mixture contained 10X PCR Buffer with MgCl2 (#D0071 Biotools, B&M Labs, Madrid, Spain), 10 mM dNTP mix (#Y02256 Invitrogen), forward and reverse primers (#67309947 y #67309948 IDT, San José, CA, USA), Taq DNA polymerase (5U/μL, #600682-51 Paq 5000 Stratagene, La Jolla, CA, USA), biotynilated dUTPs (#R0081, Thermo Scientific; 1 mM, 50 nmol) and PCR water (#37080 Bioline, London, UK) (Table 1). Reaction temperatures and times were set up: a single cycle at 94°C for 2 min; and 35 cycles including 94°C for 30 s, 60°C for 45 s and 72°C for 1 min; finally, a single cycle of 72°C for 10 min. Once finished, coverslip was softly extracted and washed carefully to eliminate the mineral oil. PCR product was fixed with 4% PFA for 10 min to prevent diffusion.

### Detection of expression

#### Immunofluorescence

A double immunofluorescence protocol was designed to detect biotin of biotinylated dUTPs incorporated during the PCR, simultaneously with vimentin protein. After amplicon fixation with PFA, sections were washed several times with 0.1 M PB and unspecific protein binding was blocked with normal goat serum (NGS 1:10; G9023, Sigma) for 1 h. Then, primary antibodies were added {mouse anti-biotin (#200-002-211 Jackson ImmunoResearch Laboratories Inc., West Grove, PA, USA; dilution 1:200 in blocking solution) and chicken anti-vimentin protein (#A73159 Abcam, Cambridge, UK; dilution 1:200 in blocking solution)} and incubated overnight at 4°C. The next day, after three washes with 0.1 M PB, secondary antibodies were added [goat anti-chicken alexa 488, (FITC), #A11039, Invitrogen] and donkey anti-mouse (alexa 647, #A31571, Invitrogen)}, both diluted 1:500 in blocking solution. DAPI was used as nuclear staining. Images were captured with a confocal microscope (FV1000; Olympus, Japan).

#### Immunogold labeling

Sections were immersed in cryoprotectant solution for 30 min (25% sucrose, in 0.1 M PB) under mild agitation at room temperature (RT). Then sections were frozen in 2-methylbuthane (#270342, Sigma) on dry ice (−60°C) for a few seconds and thawed in the cryoprotectant solution. This step was repeated 4 to 5 times and sections were transferred into ice-cold 0.1 M PB. Then, sections were blocked with blocking solution consisting in 0.3% BSAc [#25557 Aurion, Electron Microscopy Science (EMS), Hatfield Pennsylvania, USA] diluted in 0.1 M PB for 1 h at RT. After blocking, samples were incubated in mouse anti-biotin primary antibody solution (antibody diluted 1:100 in blocking solution) for 36–60 h under mild agitation at 4°C. Then samples were washed three times, 10 min each, with 0.1 M PB to remove primary antibody. From this step, all incubations were performed in the dark. Blocking was performed for 1 h at RT with a solution of 0.5% BSAc and 0.1% fish skin gelatin (#900.033, Aurion) diluted in 0.1 M PB. Samples were then incubated with gold-conjugated goat anti-mouse antibody (UltraSmall; #25121, Aurion) diluted 1:50 in the same blocking solution under mild agitation at RT. As this solution is very sensitive to light, this incubation must be kept in the dark. The next day, samples were rinsed three times with 0.1 M PB. Samples were immersed in sodium acetate solution [#S7670 Sigma, 2% sodium acetate (C2H3O2Na · 3H20) diluted in double distilled water] three times for 15 min each to finally perform silver enhancement using silver enhancement solution [mixing equal parts of developer and enhancer reagents in a dark chamber immediately before use (#25520 Silver enhancement kit, Silver enhancement R-gent SE-LM, Aurion)]. The signal increases within 10–20 min but samples have to be checked under a light microscope at 10-min intervals. To stop the reaction, samples were washed in sodium acetate solution and then incubated in gold toning solution 30 min at RT {[0.05% Gold chloride (AuCl); #HT1004 Sigma] in double distilled water}. Samples were washed in sodium thiosulfate solution [#141721,1211 Panreac, Sodium thiosulfate 0.3% (Na_2_S_2_O_3_ · 5H_2_O) in double distilled water] twice for 10 min at 4°C (sections became gray), then rinsed three times with 0.1 M PB for 10 min each at RT. Post-fixation in fixative solution of 2% GA, from an aqueous 25% stock solution, (#16210, EMS) in 0.1 M PB for 30 min at RT was the last step. Samples were kept in 0.1 M PB at 4°C.

### Conventional processing for electron microscopy

#### Post-fixation and embedding in epoxy resin

Samples were incubated in 1% osmium tetroxide OsO_4_ (#19190, EMS) in 7% glucose (in 0.1 M PB) for 30 min with gentle agitation protected from light and then washed with water three times for 5 min each at 4°C. Dehydration steps included immersions in crescent ethanol (EtOH) concentrations at 4°C (30° for 5 min, 50° for 5 min and 70° for 10 min). Samples were then exposed to 2% uranyl acetate diluted in 70° EtOH for 2 h and 30 min at 4°C. Dehydration process continued by immersion in 70° EtOH twice for 5 min, 96° EtOH twice for 10 min, 100° EtOH twice for 10 min and 100° dry EtOH (200% EtOH) for 10 min. Finally, samples were immersed carefully in propylene oxide (#8822 Baker, Bridgend, UK) at room temperature twice for 10 min and transferred them with a gentle brush to epoxy resin (araldite) (in aluminum foil molds) (araldite was prepared following manufacturers directions and vigorously shaken (#44613 Sigma Durkupan). Samples remained in the resin overnight, at room temperature on a flat surface. The next day, samples were transferred to freshly made resin between two acetate foils and resin was left to polymerize in an oven at 65°C for 72 h.

#### Ultrathin sections

After selection of the region of interest, this was pasted on a premade araldite block. We therefore obtained semithin sections (1.5 μm) with an ultramicrotome (Ultramicrotome UC6; Leica Microsystems). Under the light microscope, sections with specific signal were selected to obtain ultrathin sections (60–80 nm) and were observed at a transmission electron microscope [Phillips CM-10 (FEI), Hillsboro, OR, USA] in the Research Center Principe Felipe (CIPF) in Valencia.

## Step-by-step protocol

1. Inhibition of endogenous peroxidase (only for immunofluorescence) with 10% methanol + 10% H_2_O_2_ + 0.1 M PB in the dark ——————————15′
2. Wash with PB made in DEPC ———— 3 × 5′
3. Proteinase K digestion (5 μg/ml) at room temperature — 10′
4. Wash with PB-DEPC ———————————— 3 × 5′
5. Washes with DEPC water———————————— 2 × 1′
6. cDNA synthesis using SuperScript II RT
  6.1. Add the reagent mix to a nuclease free microcentrifuge tube (per coronal section):
    – Random primers: 3 μl
    – dNTPmix (10 mM): 3 μl
    – DEPC-H_2_O: 36 μl
  6.2. Mount sections on untreated glass slides carefully, add the previous reagent mix and cover with parafilm
  6.3. Heat sections with reagents to 65°C for 5′ and chill on ice
  6.4. Add on top:
    – 5X First Strand buffer: 12 μl
    – 0,1 M DTT: 6 μl
    – RNase OUT (40 U/ μl): 3 μl
  6.5. Incubate at 25°C————————————— 2′
  6.6. Add 1 μL (200 UI) of SuperScript II RT for each section and incubate at 25°C—10′
  6.7. Incubate at 42°C ——————————————— 50′
  6.8. Inactivate the reaction by heating at 70°C ————— 15′
7. Leave at room temperature for 20′
8. Remove parafilm and wash gently with 0.1 M PB chain reaction
9. Polymerase chain reaction
  9.1. Add the following components to a PCR tube (for a single mouse coronal section):
    - 10X PCR Buffer [200 mM Tris-HCl (ph 8.4), 500 mM KCl]: 2.5 μl
    - 10 mM dNTP mix: 0.5 μl
    - Fwd primer (10 μM): 2 μl
    - Rev primer (10 μM): 2 μl
    - Taq DNA polymerase (5 U/μL): 0.5 μl
    - dUTP biotinylated: 0.6 μl
    - Autoclaved distilled or ultrapure water: to a final volume of 25 μl
  9.2. Add mixture over the sections and cover with a round glass coverslide. Seal with a small amount of mineral oil around coverslide
  9.3. Introduce the slide in a slide-adapted thermocycler and configure 35 cycles of PCR. Use the recommended annealing and extension conditions for your Taq DNA polymerase. We used:
  – Denaturation 94°C for 2′
  – 35 cycle:
    – Step 1: Denaturation: 94°C 30″
    – Step 2: Annealing: 60°C 45″
    – Step 3: Elongation 70°C 1′
  – Final step: 70°C 10′
10. Remove mineral oil and coverslide carefully and wash profusely with 0.1 M PB
11. Fix the tissue with 4% PFA. Do this in a fumehood —— 10′
12. Wash with 0.1 M PB ————————————— 3 × 5′
13. Transfer the section carefully with a brush to a well in a 24-well or 48-well plate
14. For immunofluorescence, use primary anti-biotin antibodies with a conventional IHC protocol and fluorescent secondary antibodies. For immunogold labeling and electron microscopy, see complete protocol in Sirerol-Piquer et al., 2012; Gil-Perotin et al., 2016.

## Results

Proteolytic digestion with proteinase K was a critical step because the intensity of the signal varied with concentration, duration of digestion, temperature and at the same time, it had to be carefully controlled to avoid disruption of cellular structures (Nuovo, 1996). After testing different concentrations and temperatures, it was found that 5 μg/ml of proteinase K for 10 min at room temperature preserved the morphological structures and provided a good sensitivity. Reverse transcription to obtain cDNA was performed using random primers, and guaranteed the preservation of the RNA in the form of cDNA. The presence of genomic DNA (gDNA) in the tissue required primer design from exon/exon junctions (junctional primers) to avoid unspecific amplification of gDNA. We chose pairs of primers with similar melting temperatures and a predicted amplicon length of at least 450 bp to minimize diffusion of products through the tissue (Cmarko et al., 2014). Using specific software we confirmed that primers did not form hairpins or loops at working temperatures and we checked that primers did not amplify any region of gDNA. To rule out any homology of the amplicon with other mouse genomic regions a blast search was performed. Gradient PCR in polypropylene tubes of striatal cDNA served us to check the optimal Tm for the tissue reaction that resulted, in our case (vimentin mRNA) to be 60°C.

**Figure 2:**
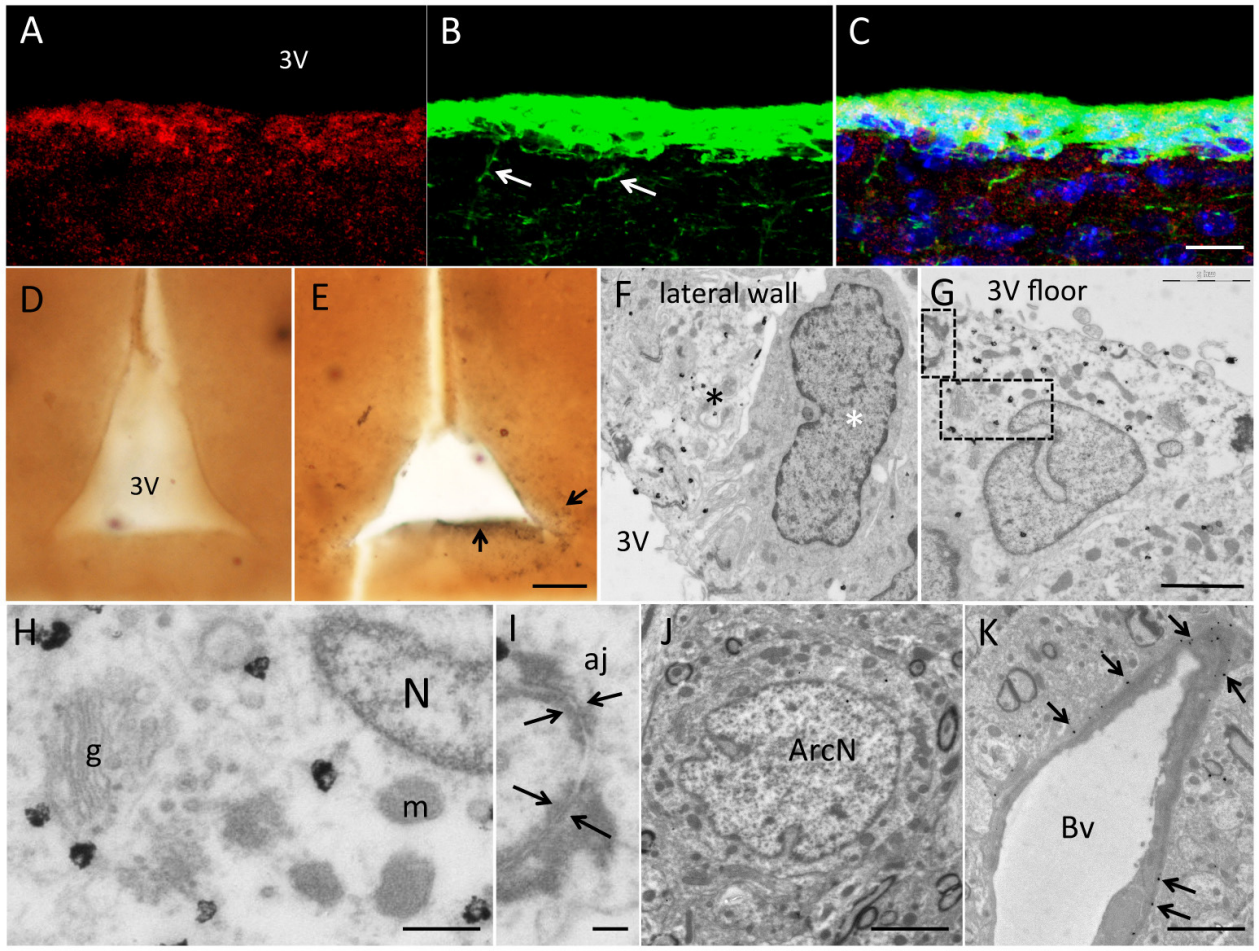
**Results of ***in situ*** RT-PCR combined with immunogold labeling for electron microscopy. (A–C)** *In situ* RT-PCR with vimentin primers and biotynilated UTP nucleotides. **(A)** Fluorescence immunohistochemistry against biotin (Cy3) **(B)** and against vimentin protein (FITC). Merged images in **(C)** showed co-localization of protein and mRNA in tanycytes, although protein was also present in basal projections (white arrows) and mRNA was confined to the cell body. **(D,E)** Low magnification pictures of 200 μm sections after *in situ* RT-PCR developed with preembedding immunogold labeling without primers (**D**, negative control) and with vimentin specific primers **(E)**. Note dark signal lining the 3V (arrows). **(F)** TEM micrograph showing gold deposition in light cells (black asterisk), compared to absence of label in darker cells (white asterisk) in the lateral 3V wall. **(G)** Positive cell in contact with the median eminence in the floor of the 3V. **(H,I)** Details of cytosol and membrane of a positive cell showing high quality images of subcellular structures such as nucleus (N), Golgi apparatus (g), mitochondria (m) **(H)** and adherent junctions (aj) (arrows in **I**). **(J)** Absence of label in mature neurons in the arcuate nucleus. **(K)** Positive label in tanycytic expansions (arrows) contacting blood vessels (Bv). Scale bars: a–c, 15 μm; d,e, 50 μm; f,g, 2,5 μm; h, 250 nm; i, 20 nm; j, 10 μm; k, 25 μm.

Under the confocal microscope, biotin staining (representing vimentin mRNA expression, Figure 2A) co-localized specifically with vimentin protein in the wall of the 3V (Figures 2B,C). Biotin signal was restricted to the cell body while protein was in the cell body but also in the basal projections (white arrows in Figure 2B), due to differential subcellular distribution. Gold particles can be seen at electron microscopy because of their high density to the electron beam. We therefore performed gold labeling with mouse anti-biotin primary antibody and colloidal gold-secondary antibodies (Figures 2D,E, Sirerol-Piquer et al., 2012; Gil-Perotin et al., 2016). Images captured at TEM (Figures 2F–K) showed gold particles within the tanycyte implying vimentin-mRNA expression. There was a clear heterogeneity regarding vimentin expression in the 3V with regions of cells devoid of gold particles, and scattered cells with a strong homogeneous signal in the lateral wall (Figure 2F), but also in the floor of the 3V (Figure 2G). Label in positive cells consisted of homogenously distributed gold particles within the cytosol, and occasional clumps of particles over cytosolic dense bodies. Tissue was highly preserved, membranes were structurally well defined, and integrity of cell-to-cell junctions and subcellular structures allowed accurate ultrastructural description (Figures 2H,I). Arcuate neurons were not labeled, because these cells did not express vimentin (Figure 2J). Blood vessel membranes, in contact with tanycytes, had their contours specifically labeled (arrows in Figure 2K). Two negative controls were paramount to test the specificity of the method. We performed PCR without primers and complete immunolabeling (Figure 2D) and PCR with primers but without primary antibody. In both assays, labeling was not detected in the brain parenchyma, except for isolated, and scarce gold particles randomly distributed considered background. We did not observe unspecific signal in the cell nuclei.

Potential pitfalls of the technique and troubleshooting are included in Table 2.

**Table 2:** **Potential pitfalls and troubleshooting**.

**Pitfalls**	**Troubleshooting**
No signal (no gold particles visualized in the tissue)	Perform RT-PCR on brain cDNA from the region of interest to test ideal cycling conditions We recommend performing immunofluorescence with anti-biotin antibodies after *in situ* RT-PCR and before electron microscopy procedures Revise RNAse free conditions Increase protease digestion
Amplicon diffusion	Check aqueous RT-PCR product (agarose gel). Ideally, there should not be amplicon band in the gel Choose primers for 350–450 bp amplicon lengths Use cross-linking fixative agents Perform more gentle proteinase digestion (reduce time or temperature) Use bulky PCR products (biotinylated dNTPs are preferable) Reduce the number of PCR cycles
Unspecific signal	We recommend to perform double immunohistochemistry with antibodies against the protein, when available Avoid non-junctional primers to minimize nuclear unspecific signal Always use negative controls at least: 1) RTPCR without primers and 2) immunohistochemistry without anti-biotin antibody) Reduce proteinase digestion (time or temperature)
Tissue deterioration	Avoid detergents Use 0.5% glutaraldehyde in the fixation mix Check thicker sections Reduce proteinase digestion (time or temperature)
Mispriming	Optimization of pH and Mg^2+^ concentrations on PCR Design highly specific oligonucleotide primers for PCR Avoid primers with secondary structures as hairpins or loops at working temperatures

## Discussion

To date, *in situ* RT-PCR is considered a complex, non-reproducible and aggressive method against cellular structures. In this work, we have adjusted the protocol to simplify its application, guaranteeing its reproducibility and preserving the ultrastructural integrity of the tissue. Hence, this optimized method combines the sensitivity of the PCR, the specificity of the *in situ* hybridization and the detail of electron microscopy.

*In situ* PCR quality is very dependent on tissue fixation. While ethanol- or acetone-fixed tissues are not suitable due to problems of DNA preservation and amplicon diffusion, formalin, and other aldehyde agents are recommended as fixative agents (Nuovo, 1996). As Nuovo explains, cross-linking agents constitute an “amplicon migration barrier,” a three-dimensional matrix of proteins and nucleic acids. The target template is fixed in the cytoplasm (in this case, RNA) with no detection of the amplicon in the aqueous amplification solution. Biotynylated nucleotides are shown to prevent amplicon diffusion because the amplicon increases its volume (Morel et al., 1998; Lassner et al., 2013). Amplicon length has also been related to diffusion and lengths between 150 and 500 bp are recommended (Morel et al., 1998; Bagasra and Harris, 2006). In our protocol we have optimized amplicon length to be around 450 bp.

We have acknowledged that permeabilization of the tissue with proteinase K is a key step in this protocol. The pore size of the amplicon migration barrier can be modified by protease digestion, so reagents are able to penetrating into the tissue, but at the same time a careful set up of exposure conditions is required to avoid deterioration of cell membranes. After testing distinct thickness of the sections, time and temperature of incubation, we determined the ideal conditions to guarantee a good ratio between intensity of label and tissue preservation to achieve ultrastructural quality standards. We did not use DNAse, and therefore avoided related tissue damage, because we used junctional primers. We extract from our results that section thickness is critical to resist enzymatic digestion, extreme temperatures during PCR, freezing with methylbutane, and finally, embedding into epoxy resin.

Embedding into resin is a necessary procedure in electron microscopy as it hardens the tissue before sectioning. We followed a conventional epoxy resin embedding protocol that polymerizes samples at a 65–70°C oven for 3 days. It is a relatively standard protocol. On the contrary, previous works have used acrylic resins that polymerize at cold temperatures or under ultraviolet radiation to preserve the integrity of nucleic acids, with the need of more complex embedding protocols (Le Guellec and Frappart, 1993). We demonstrate that the quality of the tissue is maintained using conventional protocols of inclusion in epoxy resin at high temperatures and that nucleic acids are preserved, allowing appropriate intensity of the label.

The main advantage of our method is the detection of specific target mRNA and, most importantly, the localization, identification and characterization of subcellular structures (junctions, organelles, cytoskeleton, synaptic structures) of the cell type expressing the mRNA. If we compare this method with others that are used to detect nucleic acids, such as *in situ* hybridization, the main advantages are the higher sensitivity, the simpler primer design and lower cost against the high cost and complexity of design, synthesis and/or commercially acquisition of RNA probes. In addition, *in situ* hybridization requires adjustment of stringency and hybridization conditions for each probe. All these reasons make *in situ* hybridization a complex technique, difficult to set up or reproduce. If hybridization precedes embedding, it could alter the ultrastructural morphology of the tissue and, if is carried out after embedding, the probe may not bind to nucleic acids that might have been altered. The proposed procedure is also useful if primary antibody of the protein of interest is not available (with fewer costs than generation of the antibody) or if we doubt the specificity of an antibody, since we can analyze the co-localization of mRNA and the protein on the same sample by double immunohistochemistry.

As disadvantages of this method are: (a) the need of a thermocycler adapted for slides to perform controlled PCR (although there are several models and brands available); (b) the necessity of certain expertise in conventional electron microscopy procedures. Nevertheless, for a laboratory that has basic knowledge in electron microscopy, it is a feasible method to carry out; (c) this method is not designed for quantification of mRNA, it is essentially qualitative. The amount of gold particles within the cell might correlate to mRNA expression but it will also depend on RT-PCR conditions or tissue preservation from RNAses; (d) our protocol was performed in a relatively abundant mRNA, and we have not determined the threshold of detection. This requires an individual testing for each target gene, each pair of primers, and distinct RT-PCR conditions, especially for low expressed genes.

Considering the advantages and disadvantages mentioned, the adjustments made attempt to simplify previous protocols and have increased its utility and quality of captured images making it a powerful, relatively easy and fast tool for correlative studies.

## Author contributions

SG, LC, and BC wrote the manuscript and together with JG designed the method. LC and JC performed RT-PCR and immunodetection. MD and SG were responsible of ultrastructural analyses. JF contributed with technical assistance, and finally SG supervised the project.

### Conflict of interest statement

The authors declare that the research was conducted in the absence of any commercial or financial relationships that could be construed as a potential conflict of interest. The reviewer ST and handling Editor declared their shared affiliation, and the handling Editor states that the process nevertheless met the standards of a fair and objective review.
